# An efficient one-pot and simple multicomponent approach to the synthesis of highly functionalized furans containing dialkyl phenol

**DOI:** 10.55730/1300-0527.3649

**Published:** 2024-01-31

**Authors:** Fourogh Jalili MILANI, Mahmoud NASSIRI, Jaber SALEHZADEH

**Affiliations:** 1 Department of Marine Chemistry, Faculty of Marine Science, Chabahar Maritime University, Chabahar, Iran; 2 Department of Applied Chemistry, University of Mohaghegh Ardabili, Ardabil, Iran

**Keywords:** One-pot reaction, furans, heterocycles, multicomponent reactions, arylglyoxals

## Abstract

In this study, arylglyoxals, acetylacetone, and 2,6-dimethyl phenol or 2,6-di-tert-butyl phenol are combined to efficiently synthesize a series of 1-(4-(3,5-dialkylphenyl)-2-methyl-5-phenylfuran-3-yl) ethan-1-one derivatives in excellent yields. These reactions were carried out in acetone at reflux under catalyst-free conditions in the presence of triethylamine as a base for 3 h. NMR, FT-IR, EI-MS, and elemental studies were used to characterize the products’ structural characteristics. The present study has also several benefits, such as excellent yields and the ease of workup procedure, making it an appealing, practical, and acceptable one-pot method for producing functionalized derivatives of dialkyl furan.

## 1. Introduction

The furan ring serves as an efficient building block for many biologically active targets, as well as the central component of numerous significant polymers and natural chemicals. Several scaffolds containing furans are also preferred structures in medicinal chemistry [1–3]. Furan derivatives are natural products found in various natural sources, mostly in plants, algae, microorganisms, and structural motifs in biologically drug molecules (Figure) [4]. Furans are crucial chemical building blocks for medicines, fine chemicals, and agrochemicals due to their low viscosity and strong reactivity [5,6]. According to reports, furan derivatives exhibit a variety of biological and pharmacological actions, as well as agrochemical applications, including those for treating cancer [7], amoebic infections [8], Alzheimer’s disease [9], diabetes [10], trypanosome infections [11], malaria [12], and parasitic infections [13]. On the other hand, furans are frequently employed in organic thin-film transistors [14], organic field-effect transistors [15], organic light-emitting diodes [16], organic semiconductors [17], and luminescence [18]. These features have led to the design and development of new furan heterocycles with similar properties.

Multicomponent reactions (MCRs) are highly significant reactions in which, through a one-pot reaction, more than three different reactants are directly converted into products. This method stands out as one of the most effective ways to synthesize new heterocyclic compounds in a single step. One-pot MCRs are currently gaining traction over conventional multistep organic synthesis due to their efficiency and practicality. This is because MCRs allow for the creation of pharmacologically and medicinally active targets in a single step, thereby resulting in a wide range of molecular complexity [19–21]. These reactions provide a clean reaction profile, high yield, and atom economy while enabling the formation of new bonds in a single pot without the need for multiple stages. As a result, MCRs are becoming effective and potent instruments for both industry and academia in contemporary synthetic organic chemistry.

**Scheme 1:**
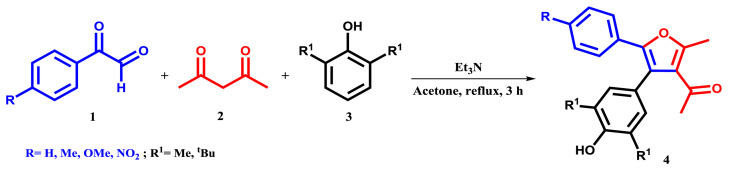
One-pot reaction for the synthesis of 1-(4-(3,5-dialkylphenyl)-2-methyl-5-phenylfuran-3-yl) ethan-1-one derivatives **4**.

Considering the role of furan derivatives in the pharmaceutical industry, their synthesis has been one of the goals of chemists. Various methods for preparing dialkyl furan derivatives have been published [22–30]. However, most of these methods suffer from one or more of the following drawbacks: the use of expensive or toxic catalyst, harsh reaction condition, multistep synthesis, the requirement of advanced starting materials, high temperature, and the need for a chromatography column to purify the products. The one-pot synthesis of 1-(4-(3,5-dialkylphenyl)-2-methyl-5-phenylfuran-3-yl) ethan-1-one derivatives (**4**) from the reactions between arylglyoxals (**1**), acetylacetone (**2**), and phenols (**3**) is reported here as a follow-up to our research on the one-pot synthesis of new functionalized furans with excellent yields (Scheme 1). In this study, we aimed to prepare dialkyl furan under more simplified conditions.

## 2. Experimental section

### 2.1. General information

All the chemicals used in this study were purchased from Merck (Darmstadt, Germany) and Fluka (Buchs, Switzerland) and were used without further purification. Melting points were determined using an Electrothermal 9100 apparatus. The mass spectra were obtained using a GCMS-QP5050A mass spectrometer operating at an ionization potential of 70 eV. Elemental analyses for C, H, and N were performed using a Heraeus CHN-O-Rapid analyzer. The ^1^H and ^13^C NMR spectra were recorded using a BRUKER DRX-250 AVANCE instrument with CDCl_3_ as the solvent and TMS as the internal standard at frequencies of 250.1 and 62.9 MHz, respectively. The IR spectra of products were measured with an FT-IR Perkin Elmer RXI.

### 2.2. General procedure for the synthesis of products (4a–h)

Arylglyoxal (1 mmol) and acetylacetone (1 mmol) were combined and agitated under reflux in acetone (10 mL) for 1 h. The reaction mixture was then supplemented with Et_3_N (1 mmol) and either 2,6-dimethyl phenol or 2,6-di-tert-butyl phenol (1 mmol) under the same conditions, and stirring was maintained for 2 h. The resulting product was a yellow solid. After the removal of the solvent, the product was rinsed with cold diethyl ether (5 mL). The residue was recrystallized from n-hexane/EtOAc 4:3 to yield **4**.

#### 1-(4-(4-hydroxy-3,5-dimethylphenyl)-2-methyl-5-phenylfuran-3-yl)ethan-1-one(4a)

Yellow powder; yield 93%; mp: 190–192 ºC. IR (KBr,*υ*, cm^−1^): 1716 (C=O). ^1^H NMR (250.1 MHz, CDCl_3_): *δ* = 2.25, 2.28, and 2.39 (9H, 3s, 3Me), 2.70 (3H, s, COMe), 5.30 (1H, s, OH), 6.82–7.81 (7H, m, 7Ar-H). ^13^C NMR (62.9 MHz, CDCl_3_): *δ* = 15.3, 19.1 and 19.3 (3Me), 30.2 (CO*Me*), 119.7, 119.8, 123.4, 123.5, 125.1, 125.3, 127.2, 128.5, 128.7, 130.3, 132.1, 132.8, 137.4 and 142.6 (14C_aro_), 149.8 and 157.3 (2C-O), 195.4 (C=O). MS (EI): *m*/*z* (%): 320 (M^+^, 8), 305 (M^+^-Me, 92), 290 (M^+^-2Me, 83), 277 (M^+^-COMe, 66), 262 (M^+^-COMe and Me, 52), 243 (M^+^-Ph, 68), 77 (Ph, 48). Anal. Calcd. For C_21_H_20_O_3_ (320.39): C, 78.73; H, 6.29. Found: C, 78.69; H, 6.23.

#### 1-(4-(4-hydroxy-3,5-dimethylphenyl)-2-methyl-5-(p-tolyl)furan-3-yl)ethan-1-one(4b)

Light yellow powder; yield 90%; mp: 196–198 ºC. IR (KBr,*υ*, cm^−1^): 1717 (C=O). ^1^H NMR (250.1 MHz, CDCl_3_): *δ* = 2.19, 2.23, 2.27 and 2.41 (12H, 4s, 4Me), 2.72 (3H, s, COMe), 5.28 (1H, s, OH), 6.83–7.80 (6H, m, 6Ar-H). ^13^C NMR (62.9 MHz, CDCl_3_): *δ* = 15.7, 19.0, 19.1 and 20.2 (4Me), 30.5 (CO*Me*), 119.4, 119.5, 124.6, 124.8, 126.3, 126.4, 127.5, 128.4, 129.5, 131.6, 134.5, 135.2, 137.7 and 143.1 (14C_aro_), 149.6 and 157.4 (2C-O), 195.2 (C=O). MS (EI): *m*/*z* (%): 334 (M^+^, 9), 319 (M^+^-Me, 86), 304 (M^+^-2Me, 64), 291 (M^+^-COMe, 82), 243 (M^+^-C_7_H_7_, 71), 228 (M^+^-C_7_H_7_ and Me, 57), 91 (C_7_H_7_, 43), and 43 (COMe, 39). Anal. Calcd. for C_22_H_22_O_3_ (334.41): C, 79.02; H, 6.63. Found: C, 78.99; H, 6.56.

#### 1-(4-(4-hydroxy-3,5-dimethylphenyl)-5-(4-methoxyphenyl)-2-methylfuran-3-yl)ethan-1-one(4c)

Yellow powder; yield 89%; mp: 185–187 ºC. IR (KBr,*υ*, cm^−1^): 1715 (C=O). ^1^H NMR (250.1 MHz, CDCl_3_): *δ* = 2.23, 2.28 and 2.38 (9H, 3s, 3Me), 2.75 (3H, s, COMe), 3.65 (3H, s, OMe), 5.29 (1H, s, OH), 6.78–7.82 (6H, m, 6Ar-H). ^13^C NMR (62.9 MHz, CDCl_3_): *δ* = 16.0, 19.3 and 19.4 (3Me), 30.1 (CO*Me*), 53.4 (OMe), 120.2, 120.3, 124.4, 124.5, 125.8, 125.9, 127.9, 128.7, 129.3, 131.3, 133.5, 135.4, 140.2 and 143.1 (14 C_aro_), 148.9 and 157.6 (2C-O), 195.4 (C=O). MS (EI): *m*/*z* (%): 350 (M^+^, 8), 335 (M^+^-Me, 77), 319 (M^+^-OMe, 90), 307 (M^+^-COMe, 67), 292 (M^+^-COMe and Me, 59), 243 (M^+^-C_7_H_7_O, 63), 107 (C_7_H_7_O, 44). Anal. Calcd. for C_22_H_22_O_4_ (350.41): C, 75.41; H, 6.33. Found: C, 75.46; H, 6.28.

#### 1-(4-(4-hydroxy-3,5-dimethylphenyl)-2-methyl-5-(4-nitrophenyl)furan-3-yl)ethan-1-one(4d)

Pale yellow powder; yield 92%; mp: 205–207 ºC. IR (KBr,*υ*, cm^−1^): 1716 (C=O). ^1^H NMR (250.1 MHz, CDCl_3_): *δ* = 2.23, 2.28 and 2.38 (9H, 3s, 3Me), 2.75 (3H, s, COMe), 5.29 (1H, s, OH), 6.78–7.85 (6H, m, 6Ar-H). ^13^C NMR (62.9 MHz, CDCl_3_): *δ* = 15.7, 19.1 and 19.3 (3Me), 30.3 (CO*Me*), 120.8, 121.0, 123.3,123.4, 126.5, 126.7, 128.7, 129.6, 132.3, 134.7, 136.3, 138.6, 141.4 and 143.7 (14 C_aro_), 149.6 and 158.1 (2C-O), 195.7 (C=O). MS (EI): *m*/*z* (%): 365 (M^+^, 7), 350 (M^+^-Me, 92), 322 (M^+^-COMe, 68), 307 (M^+^-COMe and Me, 79), 292 (M^+^-COMe and 2Me, 37), 243 (M^+^-C_6_H_4_NO_2_, 51), 122 (C_6_H_4_NO_2_, 33). Anal. Calcd. for C_21_H_19_NO_5_ (365.38): C, 69.03; H, 5.24; N, 3.83. Found: C, 68.92; H, 5.30; N, 3.88.

#### 1-(4-(3,5-di-tert-butyl-4-hydroxyphenyl)-2-methyl-5-phenylfuran-3-yl)ethan-1-one(4e)

Lightyellow powder; yield 94%; mp: 194–196 ºC. IR (KBr,*υ*, cm^−1^): 1722 (C=O). ^1^H NMR (250.1MHz, CDCl_3_): *δ* = 1.40 and 1.42 (18H, 2s, 2C*Me_3_*), 2.40 (3H, s, Me), 2.70 (3H, s, COMe), 5.19 (1H, s, OH), 6.83–7.79 (7H, m, 7Ar-H). ^13^C NMR (62.9 MHz, CDCl_3_): *δ* = 14.9 (3H, s, Me), 29.4, 30.3 and 31.2 (2C*Me_3_* and CO*Me*), 33.8 and 34.0 (2*C*Me_3_), 121.3, 121.5, 124.2, 124.3, 125.9, 126.0,128.5, 130.7, 131.3, 135.8, 136.1, 137.9, 138.2, 140.6 and 144.5 (14 C_aro_), 147.8 and 157.4 (2C-O), 196.1 (C=O).MS (EI): *m*/*z* (%): 404 (M^+^, 8), 361 (M^+^-COMe, 76), 347 (M^+^-CMe_3_, 83), 332 (M^+^-CMe_3_ and Me, 55), 327 (M^+^-Ph, 59), 57 (CMe_3_, 40). Anal. Calcd. for C_27_H_32_O_3_ (404.55): C, 80.16; H, 7.97. Found: C, 80.10; H, 7.94.

#### 1-(4-(3,5-di-tert-butyl-4-hydroxyphenyl)-2-methyl-5-(p-tolyl)furan-3-yl)ethan-1-one(4f)

Yellow powder; yield 91%; mp: 201–203 ºC. IR (KBr,*υ*, cm^−1^): 1720 (C=O). ^1^H NMR (250.1 MHz, CDCl_3_): *δ* = 1.38 and 1.39 (18H, 2s, 2C*Me_3_*), 2.23 and 2.40 (6H, 2s, 2Me), 2.71 (3H, s, COMe), 5.22 (1H, s, OH), 6.83–7.76 (6H, m, 6Ar-H). ^13^C NMR (62.9 MHz, CDCl_3_): *δ* = 15.3 (3H, s, Me), 22.3 (Me), 29.8, 30.5 and 31.6 (2C*Me_3_* and CO*Me*), 33.6 and 33.7 (2*C*Me_3_), 120.2, 120.3, 123.5, 123.6, 126.1, 126.3, 127.9, 128.4, 132.1, 134.7, 136.3, 139.6, 142.2 and 144.3 (14 C_aro_), 147.6 and 157.5 (2C-O), 195.9 (C=O). MS (EI): *m*/*z* (%): 418 (M^+^, 9), 403 (M^+^-Me, 90), 375 (M^+^-COMe, 81), 360 (M^+^-COMe and Me, 73), 361 (M^+^-CMe_3_, 78), 346 (M^+^-CMe_3_ and Me, 58), 91 (C_7_H_7_, 42), 57 (CMe_3_, 44), 43 (COMe, 37). Anal. Calcd. for C_28_H_34_O_3_ (418.58): C, 80.35; H, 8.19. Found: C, 80.26; H, 8.19.

#### 1-(4-(3,5-di-tert-butyl-4-hydroxyphenyl)-5-(4-methoxyphenyl)-2-methylfuran-3-yl)ethan-1-one(4g)

Yellow powder; yield 91%; mp: 197–199 ºC. IR (KBr,*υ*, cm^−1^): 1723 (C=O). ^1^H NMR (250.1 MHz, CDCl_3_): *δ* = 1.36 and 1.38 (18H, 2s, 2C*Me_3_*), 2.39 (3H, s, Me), 2.69 (3H, s, COMe), 3.64 (3H, s, OMe), 5.25 (1H, s, OH), 6.81–7.82 (6H, m, 6Ar-H). ^13^C NMR (62.9 MHz, CDCl_3_): *δ* = 15.6 (3H, s, Me), 30.2, 30.5 and 31.4 (2C*Me_3_* and CO*Me*), 33.4 and 33.5 (2*C*Me_3_), 52.8 (OMe), 119.8, 120.0, 122.4, 122.5, 125.2, 125.3, 128.0, 132.0, 134.5, 137.7, 138.1, 141.2, 143.6, and 145.8 (14 C_aro_), 148.4 and 157.3 (2C-O), 196.0 (C=O). MS (EI): *m*/*z* (%): 434 (M^+^, 7), 419 (M^+^-Me, 91), 377 (M^+^-CMe_3_, 88), 360 (M^+^-COMe and OMe, 62), 346 (M^+^-CMe_3_ and OMe, 67), 327 (M^+^-C_7_H_7_O, 61), 107 (C_7_H_7_O, 42), 57 (CMe_3_, 48), 43 (COMe, 33). Anal. Calcd. for C_28_H_34_O_4_ (434.58): C, 77.39; H, 7.89. Found: C, 77.36; H, 7.84.

#### 1-(4-(3,5-di-tert-butyl-4-hydroxyphenyl)-2-methyl-5-(4-nitrophenyl)furan-3-yl)ethan-1-one (4h)

Pale yellow powder; yield 93%; mp: 209–211 ºC. IR (KBr,*υ*, cm^−1^): 1723 (C=O). ^1^H NMR (250.1 MHz, CDCl_3_): *δ* = 1.39 and 1.40 (18H, 2s, 2C*Me_3_*), 2.42 (3H, s, Me), 2.73 (3H, s, COMe), 5.25 (1H, s, OH), 6.80–7.84 (6H, m, 6Ar-H). ^13^C NMR (62.9 MHz, CDCl_3_): *δ* = 15.9 (3H, s, Me), 30.2, 30.6, and 31.3 (2C*Me_3_* and CO*Me*), 33.7 and 33.8 (2*C*Me_3_), 121.6, 121.7, 123.2, 123.3, 125.8, 125.9, 128.5, 129.7, 133.9, 137.8, 140.3, 143.9, 144.4 and 146.7 (14 C_aro_), 148.2 and 158.1 (2C-O), 196.2 (C=O). MS (EI): *m*/*z* (%): 449 (M^+^, 7), 406 (M^+^-COMe, 83), 392 (M^+^-CMe_3_, 75), 377 (M^+^-CMe_3_ and Me, 72), 327 (M^+^-C_6_H_4_NO_2_, 84), 284 (M^+^-C_6_H_4_NO_2_ and COMe, 43), 270 (M^+^-C_6_H_4_NO_2_ and CMe_3_, 51), 122 (C_6_H_4_NO_2_, 45), 57 (CMe_3_, 48). Anal. Calcd. for C_27_H_31_NO_5_ (449.54): C, 72.14; H, 6.95; N, 3.11. Found: C, 72.27; H, 7.02; N, 3.06.

## 3. Results and discussion

### 3.1. Synthesis and optimization of reaction conditions

As a model reaction for the synthesis of 1-(4-(4-hydroxy-3,5-dimethylphenyl)-2-methyl-5-phenyl furan-3-yl) ethan-1-one **4a**, the one-pot reaction between arylglyoxals (**1**), acetylacetone (**2**), and 2,6-dimethylphenol (**3**) was selected (Table 1). The initial stages of the reaction occurred in water with one equimolar of Et_3_N present at room temperature. TLC was utilized to monitor the progress of the reaction. Upon compilation of the reaction, product **4a** was isolated by filtration, yielding an orange powder. The reaction yield was 40%. In order to optimize the reaction conditions, the reaction was conducted in the presence of various bases and solvents. The results were presented in Table 1. As shown in Table 1, in the presence of 1,4-diazabicyclo [2.2.2] octane (DABCO), the reaction yield was 30% (Table 1, entries 1–5). The presence of KOH and K_2_CO_3_ in water resulted in poor reaction yields, and pyridine in water did not facilitate the reaction. Consequently, Et_3_N was selected as the proper base for this reaction. The reaction yield could not be increased when using EtOH and CH_2_Cl_2_ as solvents (Table 1, entries 6 and 7). Although the reaction yield increased in acetone, as well as in DMSO or CH_3_CN (Table 1, entries 8–10), the rise in acetone was more significant. Further research on the effect of temperature on reaction yield revealed that the reaction yield was higher when carried out in refluxing acetone (Table 1, entry 11). Therefore, the optimal temperature for the synthesis of 1-(4-(4-hydroxy-3,5-dimethylphenyl)-2-methyl-5-phenylfuran-3-yl) ethan-1-one 4a is determined to be refluxing. Additionally, employing more Et_3_N did not impact the reaction yield, indicating that one equimolar of Et_3_N is the ideal amount of base for this reaction (Table 1, entries 11 and 12). In this reaction, no detectable byproducts were formed. Along with the desired product, small amounts of acetone-soluble dark materials were formed, which were separated from the main product by filtration.

### 3.2. Characterization of products

The structures of products were confirmed using FT-IR, ^1^H- and ^13^C NMR spectra, elemental analysis, and mass spectroscopic data. For example, the ^1^H NMR spectrum of **4a** exhibited five singlets at 2.25, 2.28, 2.39, and 2.70 ppm (12H, 4Me), and 5.30 ppm (1H, OH). Additionally, aromatic protons were observed as multiplets at 6.82–7.81 ppm (7Ar-H) for the phenyl and phenol moieties [31–33]. The hypothesized structure is supported by the ^13^C NMR spectrum of **4a**, which displayed 21 different resonances. Additionally, product **4a** exhibited ^13^C NMR resonances for the 4Me, 2C-O, and Me-C=O carbons at 15.3, 19.1, 19.3, 30.2, 149.8, and 157.3 ppm, respectively [34]. The ^1^H NMR and ^13^C NMR spectra of **4b-h** are similar to those of **4a**. The FT-IR spectrum of compound **4a** showed an absorption band attributable to the carbonyl group at 1716 cm^−1^. The mass spectrum of this compound displayed the molecular ion peak at 320 *m*/*z*, which is consistent with the proposed structure.

The one-pot reaction of arylglyoxals (**1**), acetylacetone (**2**), and 2,6-dimethylphenol (**3**) in the presence of Et_3_N in acetone solvent yielded compounds **4a–h**. The reactions exhibited high efficiency, and within 3 h, the products **4a–h** were obtained with high yields (Table 2).

**Scheme 2:**
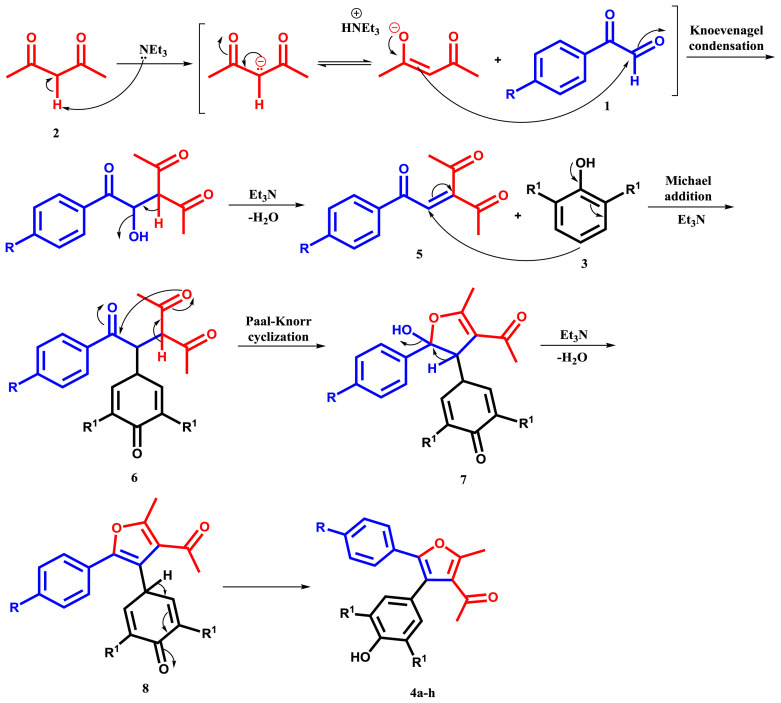
The proposed mechanism for the preparation of compounds **4a–h**.

In Scheme 2, the suggested mechanism for this reaction is depicted. Although the mechanistic specifics of the reaction are unknown, a credible explanation for the generation of product can be proposed. Initially, under reflux conditions for 1 h, arylglyoxal (**1**) and acetylacetone (**2**) undergo condensation by the Knoevenagel reaction to yield intermediate (**5**). Next, the Michael addition of phenol (**3**) to the intermediate (**5**) produces reactive 1,4-diketone (**6**). Triethylamine was used as a base during this stage, and the mixture was agitated under identical conditions for 2 h. The Paal-Knorr cyclization of the given 1,4-diketone yielded (**8**). Ultimately, this intermediate (**8**) is converted into product (**4**) via a formal [1,5] hydrogen shift [35].

## 4. Conclusion

The research was one of the first to use derivatives of high-function dialkyl furan instead of diene for a simple and easy one-pot synthesis method. In this method, the reaction between arylglyoxals, acetylacetone, 2,6-dimethyl phenol, or 2,6-di-tert-butyl phenol under reflux in the presence of triethylamine was used to yield 1-(4-(3,5-dialkylphenyl)-2-methyl-5-phenylfuran-3-yl) ethan-1-one derivatives in excellent yields. The advantages of the present method include a straightforward procedure, an easy workup, a quick reaction time, readily accessible starting ingredients, and simple purification of products without using a chromatography column, which make it a new alternative route to other dialkyl furan syntheses.

## Figures and Tables

**Figure:**
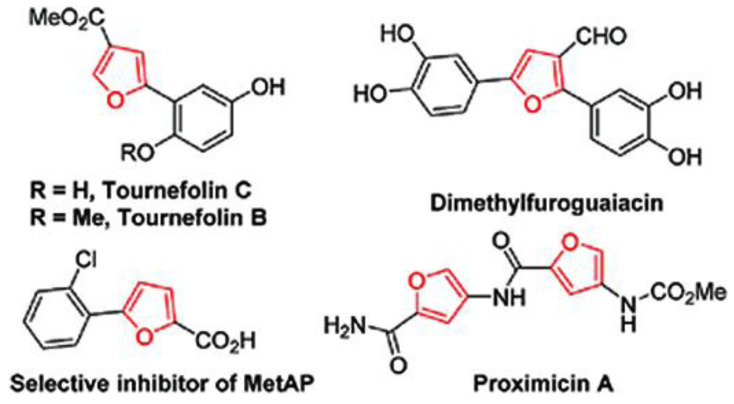
Furan-derived natural products and drugs.

**Table 1:** Optimization of the reaction conditions for the synthesis of compound **4a**.

Entry	Solvent	Base (mol%)	T (°C)	Time (h)	Yield% of **4a**[a]
1	H_2_O	Et_3_N (100)	RT	24	40
2	H_2_O	KOH (100)	RT	12	Trace
3	H_2_O	K_2_CO_3_ (100)	RT	12	Trace
4	H_2_O	Pyridine (100)	RT	12	N.R.
5	H_2_O	DABCO (100)	RT	12	30
6	EtOH	Et_3_N (100)	RT	24	40
7	CH_2_Cl_2_	Et_3_N (100)	RT	24	20
8	DMSO	Et_3_N (100)	RT	24	33
9	CH_3_CN	Et_3_N (100)	RT	8	51
10	Acetone	Et_3_N (100)	RT	6	64
11	Acetone	Et_3_N (100)	Reflux	3	93
12	Acetone	Et_3_N (150)	Reflux	3	93

[a] Isolated yields

RT: room temperature.

**Table 2:** One-pot synthesis of 1-(4-(3,5-dialkylphenyl)-2-methyl-5-phenylfuran-3-yl) ethan-1-one derivatives **4a–h**.

Entry	R	R^1^	Product	Yield (%)[a]
1	H	Me	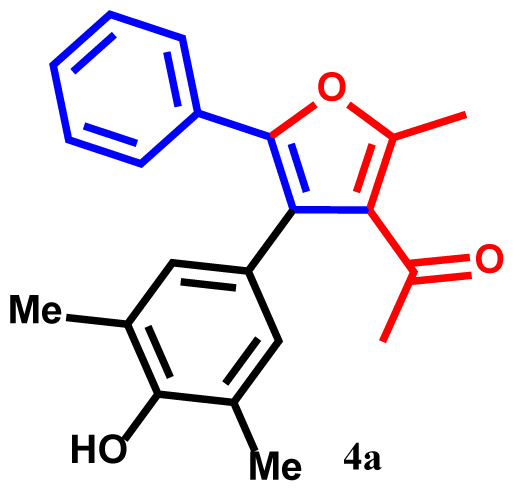	93
2	Me	Me	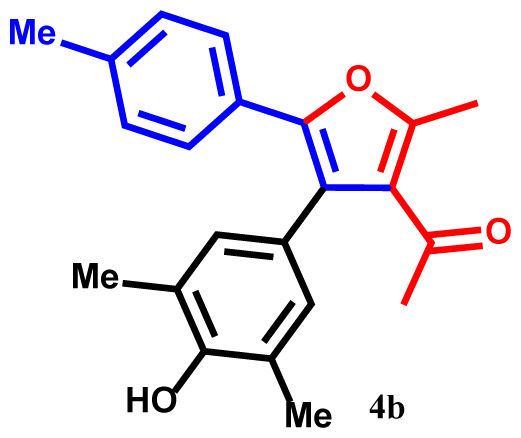	90
3	OMe	Me	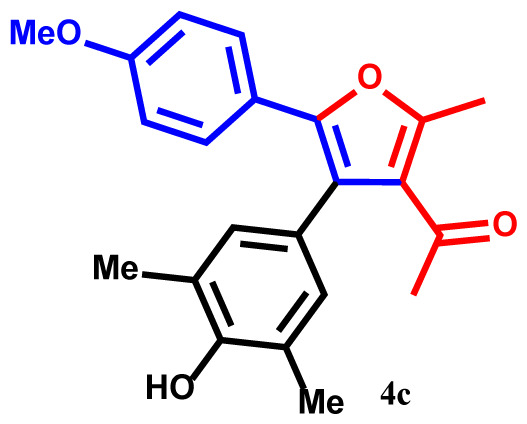	89
4	NO_2_	Me	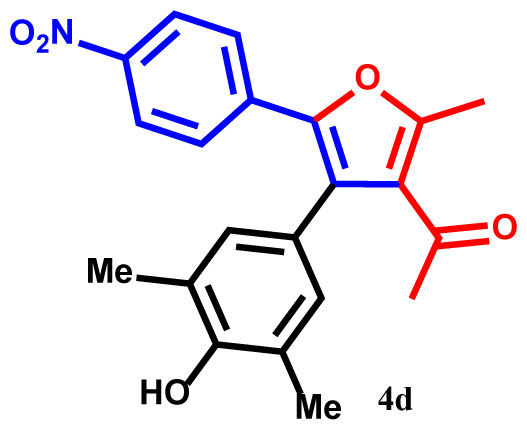	92
5	H	^t^Bu	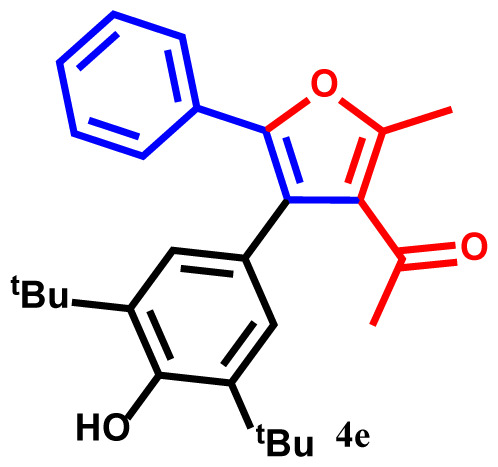	94
6	Me	^t^Bu	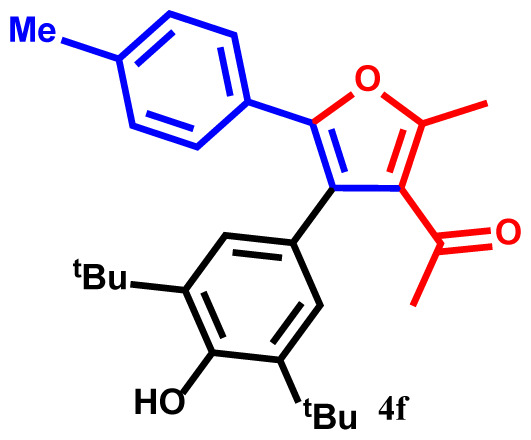	91
7	OMe	^t^Bu	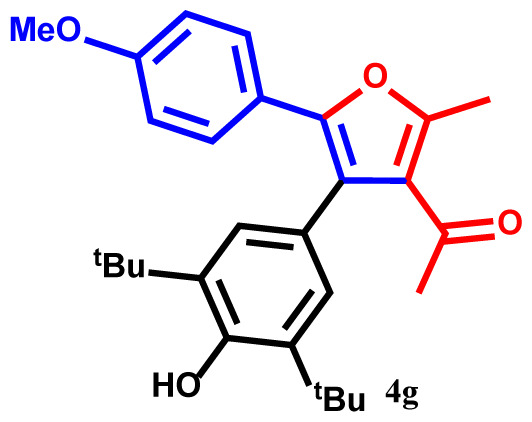	91
8	NO_2_	^t^Bu	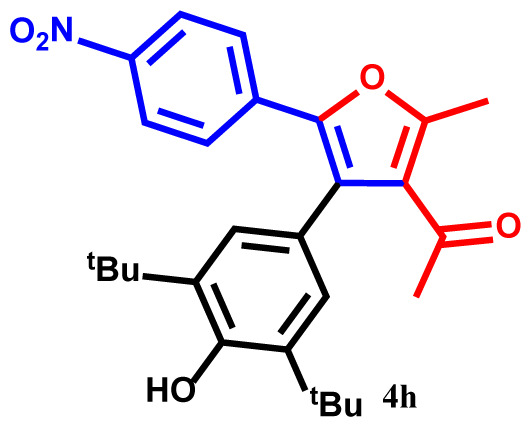	93

[a] Isolated yields.

## References

[1] Vranova J, Ciesarova Z (2009). Furan in food-a review. Czech Journal of Food Sciences.

[2] Emektar K, Kantekin Erdogan MN, Tekin A (2022). Furan formation in some vegetable oils during heat treatments. Food Chemistry.

[3] Saeed A, Ashraf S, Shabir G, Hökelek T, Floerke U (2022). Synthesis, X-Ray crystallography and HF/DFT analysis of N (diethylcarbamothioyl) furan-2-carboxamide, analyzed by experimental and theoretical methods. Journal of Molecular Structure.

[4] Ye QZ, Xie SX, Huang M, Huang WJ, Lu JP (2004). Metalloform-Selective Inhibitors of Escherichia c oli Methionine Aminopeptidase and X-ray Structure of a Mn (II)-Form Enzyme Complexed with an Inhibitor. Journal of the American Chemical Society.

[5] Liu J, Li F, Zheng X, Su J, Yu Y (2021). Lipase-catalyzed synthesis of polyhydroxyalkyl furans from unprotected sugars and malononitrile. Process Biochemistry.

[6] Güzel E, Şaki N, Akın M, Nebioğlu M, Şişman İ (2018). Zinc and chloroindium complexes of furan-2-ylmethoxy substituted phthalocyanines: Preparation and investigation of aggregation, singlet oxygen generation, antioxidant and antimicrobial properties. Synthetic Metals.

[7] Sun M, Wan Q, Ding MW (2019). New facile synthesis of furan-2 (3H)-ones and 2, 3, 5-trisubstituted furans via intramolecular Wittig reaction of acid anhydride. Tetrahedron.

[8] Ansari MF, Siddiqui SM, Ahmad K, Avecilla F, Dharavath S (2016). Synthesis, antiamoebic and molecular docking studies of furan-thiazolidinone hybrids. European Journal of Medicinal Chemistry.

[9] Kumar N, Gusain A, Kumar J, Singh R, Hota PK (2021). Anti-oxidation properties of 2-substituted furan derivatives: A mechanistic study. Journal of Luminescence.

[10] Mphahlele MJ, Choong YS, Maluleka MM, Gildenhuys S (2020). Synthesis, in vitro evaluation and molecular docking of the 5-acetyl-2-aryl-6-hydroxybenzo [b] furans against multiple targets linked to type 2 diabetes. Biomolecules.

[11] Albayati MR, Kansız S, Lgaz H, Kaya S, Dege N (2020). Synthesis, experimental and theoretical characterization of (E)-2-((2, 3-dimethylphenyl) amino)-N′-(furan-2-ylmethylene) benzohydrazide. Journal of Molecular Structure.

[12] Cui Z, Li Y, Ling Y, Huang J, Cui J (2010). New class of potent antitumor acylhydrazone derivatives containing furan. European Journal of Medicinal Chemistry.

[13] Gomonov KA, Pelipko VV, Litvinov IA, Baichurin RI, Makarenko SV (2023). Synthesis of substituted furan-3-carboxylates from alkyl 3-bromo-3-nitroacrylates. Mendeleev Communications.

[14] Hendsbee AD, Sun JP, McCormick TM, Hill IG, Welch GC (2015). Unusual loss of electron mobility upon furan for thiophene substitution in a molecular semiconductor. Organic Electronics.

[15] Ando S, Nishida JI, Fujiwara E, Tada H, Inoue Y (2006). Physical properties and field-effect transistors based on novel thiazolothiazole/heterocyclic and thiazolothiazole/phenylene co-oligomers. Synthetic Metals.

[16] Hao W, Zou S, Gao J, Zhang H, Chen R (2018). Organic single-crystalline transistors based on Benzo [b] thiophen-Benzo [b] furan analogues with contorted configuration. Organic Electronics.

[17] Kazantsev MS, Beloborodova AA, Kuimov AD, Koskin IP, Frantseva ES (2018). Synthesis, luminescence and charge transport properties of furan/phenylene co-oligomers: The study of conjugation length effect. Organic Electronics.

[18] Heidari M, Anary Abbasinejad M, Ghazanfari D, Akhgar MR (2018). Synthesis of Polyfunctionalized Furans by Three-component Reaction Between Arylglyoxals, 1, 3-Diketones and Isocyanides. Letters in Organic Chemistry.

[19] Biggs Houck JE, Younai A, Shaw JT (2010). Recent advances in multicomponent reactions for diversity-oriented synthesis. Current Opinion in Chemical Biology.

[20] Orru R, Ruijter E (2010). Synthesis of heterocycles via multicomponent reactions I. Topics in Heterocyclic Chemistry.

[21] Komogortsev AN, Lichitsky BV, Melekhina VG (2021). Multicomponent Approach to the Synthesis of 4-(1 H-indol-3-yl)-5-(4-methoxyphenyl) furan-2 (5 H)-one. Molbank.

[22] Chiurchiù E, Gabrielli S, Ballini R, Palmieri A (2019). A new valuable synthesis of polyfunctionalized furans starting from β-nitroenones and active methylene compounds. Molecules.

[23] Komeyama K, Ohama Y, Takaki K (2011). Direct synthesis of highly substituted furans from acyloins and active methylene compounds catalyzed by bismuth triflate. Chemistry Letters.

[24] Doostmohammadi R, Hazeri N (2013). Application of silica gel-supported polyphosphoric acid (PPA/SiO2) as a reusable solid acid catalyst for one-pot multi-component synthesis of 3, 4, 5-substituted furan-2 (5H)-ones. Letters in Organic Chemistry.

[25] Tekale SU, Kauthale SS, Pagore VP, Jadhav VB, Pawar RP (2013). ZnO nanoparticle-catalyzed efficient one-pot three-component synthesis of 3, 4, 5-trisubstituted furan-2 (5H)-ones. Journal of the Iranian Chemical Society.

[26] Ghasemnejad Bosra H, Faraje M, Habibzadeh S, Ramzanian Lehmali F (2010). An efficient one-pot synthesis of highly substituted furans catalyzed by N-bromosuccinimide. Journal of the Serbian Chemical Society.

[27] Dehbandia B, Shafieeb S (2019). Synthesis of functionalized furan using multicomponent reaction of isatin. Iranian Journal of Organic Chemistry.

[28] Wang Y, Czabala P, Raj M (2023). Bioinspired one-pot furan-thiol-amine multicomponent reaction for making heterocycles and its applications. Nature Communications.

[29] Maghsoodlou MT, Khorassani SM, Hazeri N, Heydari R, Marandi G (2006). The new γ-spiroiminolactone synthesis by reaction between alkyl or aryl isocyanides and 1, 10-phenanthroline-5, 6-dione in the presence of acetylenic esters. Journal of Chemical Research.

[30] Doostmohammadi R, Maghsoodlou MT, Habibi Khorassani SM (2012). Formic acid as an efficient catalyst for the one-pot preparation of furan-2 (5H)-ones under solvent-free condition. Iranian Journal of Organic Chemistry.

[31] Dehghanzadeh F, Shahrokhabadi F, Anary Abbasinejad M (2019). A simple route for synthesis of 5-(furan-3-yl) barbiturate/thiobarbiturate derivatives via a multi-component reaction between arylglyoxals, acetylacetone and barbituric/thiobarbituric acid. Arkivoc.

[32] Latifi M, Talebdizaeh M, Anary Abbasinejad M (2018). Reactions of cyclohexyl isocyanide, dialkyl acetylenedicarboxylates and 1-aryl-2-ene-3-acetyl-1, 4-diketones: one-pot synthesis of highly functionalized 5-cyclohexylimino-2, 5-dihydrofurans. Arkivoc.

[33] Mehrabi H, Dastouri F, Asadi S, Alizadeh Bami F, Ranjbar Karimi R (2020). One-pot, regioselective synthesis of functionalized indole derivatives: a three-component domino reaction of arylamine, arylglyoxal, and 4-hydroxycoumarin or 4-hydroxy-6-methyl-2-pyrone. Arkivoc.

[34] Breitmaier E, Voelter W (1986). Carbon-13 NMR Spectroscopy: Methods and Applications İnorganic Chemistry.

[35] Yavari I, Anary Abbasinejad M, Alizadeh A (2002). A novel approach to the synthesis of highly functionalized furans. Tetrahedron Letters.

